# Biological Monitoring of Exposure to Benzene in Port Workers

**DOI:** 10.3389/fpubh.2020.00271

**Published:** 2020-07-17

**Authors:** Luigi De Maria, Caterina Ledda, Antonio Caputi, Francesca Mansi, Enza Sabrina Silvana Cannone, Stefania Sponselli, Domenica Cavone, Francesco Birtolo, Emanuele Cannizzaro, Giovanni Maria Ferri, Venerando Rapisarda, Luigi Vimercati

**Affiliations:** ^1^Interdisciplinary Department of Medicine (DIM), Section of Occupational Medicine “B.Ramazzini”, School of Medicine, University of Bari “A. Moro”, Bari, Italy; ^2^Department of Clinical and Experimental Medicine, Occupational Medicine, University of Catania, Catania, Italy; ^3^Department of Health Promotion Sciences Maternal and Infantile Care, Internal Medicine and Medical Specialities “Giuseppe D'Alessandro”, Occupational Medicine, University of Palermo, Palermo, Italy

**Keywords:** port workers, benzene, biological monitoring, occupational exposure, t, t-muconic acid

## Abstract

Port workers are exposed to a wide range of occupational hazards that can cause injuries and occupational diseases. Among these, exposure to benzene is one of the most important but least studied. The highest occupational exposures for port workers occur during the filling and loading of gasoline, and cleaning of tanks and receptacles. The aim of the study was to evaluate occupational exposure to low levels of benzene by measuring trans,trans-muconic acid (t,t-MA) in urine samples from workers operating at fuelling stations in a tourist port of Southern Italy. The overall sample was composed of 43 port workers of a tourist port in Southern Italy. In 2018, each participant provided two (morning and evening) urine samples for the determination of urinary t,t-MA. Urinary excretion of t,t-MA was always higher at the end of the work shift than at the beginning with significant difference (*p* = 0.002). In smokers, median t,t-MA urinary excretion is higher than non-smokers both at the beginning (90.5 μg/g creatinine vs. 61.45 μg/g creatinine) and at the end of the work shift (128.2 μg/g creatinine vs. 89.5 μg/g creatinine). Urinary excretion of t,t-MA is higher at the end of the work shift than at the beginning in both smokers and non-smokers, but the difference is significantly higher in non-smokers (*p* = 0.003) than in smokers (*p* = 0.05). In conclusion, our results showed that the role of inhaled benzene at fuelling stations in a tourist port can be relevant. On the basis of these results and the known adverse effects of benzene on human health, we encourage the use of personal protective equipment in the fuelling area of ports in order to minimize exposure to benzene to workers.

## Introduction

According to the European Commission, seaports play an important role for economic development by promoting the European Union's external trade (90% of the total, in terms of weight) and internal market exchanges (40% of the total) (1). In the seaports of the 22 maritime Member States of the European Union, around 110,000 port workers are engaged in the loading and unloading of ships (2).

In the same way as other working sectors, port workers are exposed to a wide range of occupational hazards that can cause injuries and occupational diseases (3–7). They have a high risk of exposure to ergonomic hazards (e.g., repetition of movements, awkward positions), biological hazards (e.g., animals, microorganisms, bacteria, viruses, and fungi), physical agents (e.g., extreme temperatures, noise, vibrations, and radiation), psychosocial hazards (fatigue, irregular working hours, shift work, etc.), and chemical substances (8, 9). Furthermore, port workers may be exposed to asbestos in the course of their work (10). For a long time, asbestos was widely used in various fields (maritime, industrial, military and construction sectors, etc.), and the unaware use of this mineral caused adverse effect on human health and the environment (11–14).

Many industrial, agricultural, and medical organizations use hazardous substances (15–17). In this field, the main operations that expose port workers to contact/inhalation of harmful chemicals are as follows: mechanical maintenance, cleaning and sanitizing of ship interiors, unloading of raw materials from the holds of ships and/or loading of finished products, storage of chemicals, storage and transport of vehicles, and refueling of ships at gasoline stations. Actually, port workers can be exposed to different types of toxic agents such as carbon monoxide, volatile organic compounds (e.g., benzene), nitrogen oxides, sulfur oxides, particulate matter, metallic elements, and pesticides. Other chemicals produced by port activities are formaldehyde, polycyclic aromatic hydrocarbons, and dioxins (18–21). Many of these have mutagenic and/or carcinogenic effects such as benzene, one of the most toxic environmental and occupational pollutants (22). Exposure to benzene usually occurs in a wide variety of occupational fields. In particular, this toxin is produced from chemical plants, oil refineries, petrochemical industries, coke production plants, hazardous waste landfills, and petrol service stations (23, 24). Benzene is also present in living environment, released by cigarette smoking and vehicles exhausting fumes (25). According to the European Chemical Agency (ECHA), the highest occupational exposures occur during the filling and loading of gasoline, and cleaning of tanks and receptacles (26). Because of its known carcinogenic properties (acute myeloid leukemia—acute non-lymphocytic leukemia), the International Agency for Research on Cancer (IARC) has classified benzene as carcinogenic for humans (Group 1) (27).

Benzene is highly volatile, and occupational exposure occurs mainly by inhalation, although dermal exposure is possible in some specific conditions such as immersion of the skin in solution or when the airborne concentration of benzene is very low (28, 29). Following exposure, benzene is partially eliminated in the exhaled air (17%); the remaining part is metabolized and excreted in the urine in the form of phenolic compounds (e.g., phenol, hydroquinone, catechol, and trihydroxybenzene), S-phenylmercapturic acid (S-PMA), trans,trans-muconic acid (t,t-MA), and unmetabolized benzene (U-benzene) (30, 31). Biological monitoring of benzene exposure involves the measurement of benzene levels or its metabolites in the biological samples. Suggested biomarkers for benzene exposure in occupational settings are urinary samples of unmetabolized benzene or S-PMA (32, 33). For the purpose of biomonitoring to low concentrations of benzene, the used biomarker is the t,t-MA. This biomarker is a urinary metabolite of benzene that is used in routine practice because it is a reliable and relatively convenient biomarker (34–36).

Italian Legislative Decrees 152/2006 and 66/2005 regulate, for the purposes of the prevention and limitation of atmospheric pollution, the characteristics of marine diesel and establish that the benzene content must be <1.0% (v/v) and that of total aromatic polycyl hydrocarbons must be lower than 35% (v/v). Italian Legislative Decree 155/2010 established the Occupational Exposure Limit (OEL) for benzene of 1.6 mg/m^3^, with a Short-Term Exposure Limit (STEL), equivalent to 15-min average exposure, of 8 mg/m^3^. The EU is preparing for a much lower OEL. In 2017, the ECHA suggested a new OEL of 0.1 ppm, or 0.3 mg m^−3^, and a year later (2018), the ECHA RAC proposed an even lower OEL of 0.05 ppm, or 0.16 mg m^−3^ (33).

For the purpose of biomonitoring to low concentrations of benzene, one of the most used biomarkers is the t,t-MA. This well-known and relatively convenient biomarker is a urinary metabolite of benzene (34–36). However, t,t-MA levels in urine are influenced by other factors such as cigarette smoking and sorbic acid (food preservative) introduced with diet (24).

The American Conference of Governmental Industrial Hygienists (ACGIH) established a Biological Exposure Index (BEI) of 500 μg t,t-MA/g creatinine in urine for occupational benzene exposure (equal to 0.75 μg/ml with respect to an excretion of 1.5 g/L creatinine in urine) (37).

Although several biomonitoring studies have been conducted to assess occupational exposure to benzene in different group of workers (23, 24, 38), there are no reports in literature on biological monitoring in port workers at gasoline stations. The aim of this study was to evaluate occupational exposure to low levels of benzene by measuring t,t-MA in urine samples from 43 workers operating at fuelling stations in a tourist port of Southern Italy.

## Materials and Methods

### Study Population

The overall sample consisted of 43 male port workers, and the selection was carried out on a random basis. The work activities consisted of refueling pleasure boats. Data regarding personal characteristics, length of service, and smoking habit were collected by technical personnel. All subjects had given written informed consent to take part in the study, after having received a full explanation of the aims and the methods.

### Biological Monitoring

The overall sample was composed of 43 port workers potentially exposed to low levels of benzene. Urine sampling was performed between April 2018 and June 2018.

Each participant provided two (morning and evening) urine samples for determination of urinary t,t-MA. The first sampling was conducted in the early morning (the first urination of the day), and the second sample was collected at the end of the 8 h work shift with a 6-day week. Urine samples were collected in 10-ml polystyrene single-use containers and were frozen at −20°C until analysis. The benzene metabolite t,t-MA is widely used as a biological indicator of exposure to this xenobiotic. The analysis of t,t-MA was performed by high-performance liquid chromatography (HPLC) with UV detection method, using a commercial laboratory kit (Chromsystems Instruments &Chemicals GmbH, Grafelfing, Germany) (39). Briefly, 750 μl of the internal standard was put into a reaction vial and mixed using a vortex. The sample (250 μl of urine) preparation is based on the efficient and selective purification with solid phase extraction. This includes the addition of an internal standard (provided by the manufacturer) to the sample with a simultaneous pH adjustment and subsequent transfer to the SPE column. Sequenced washing steps (with buffer 2 and 3 provided by the manufacturer) are then performed to eliminate interfering substances. The limit of quantification is 0.02 mg/L, the linearity is 0.02 up to 10 mg/L, the recovery is between 93% and 98%, the intra-assay coefficient of variation (CV) is <1.5–1.7%, and the interassay CV is <2.9–3.4%. Internal quality was secured by the Shewhart control cards and by the application of the Westgard Rules. The external control is assumed by the matrix analysis with known concentration.

Finally, the t,t-MA is eluted and stabilized simultaneously. This analysis method is very sensitive and allows us to determine concentrations over 20 μg/L. Urinary creatinine was also quantified to check the acceptability of each urine sample and to make appropriate corrections to some of the biomarkers measured. Urinary creatinine range, according to WHO criteria, should be between 0.3 and 3 g/L (40). Urinary creatinine concentrations were measured using a fully automated clinical chemistry analyzer (Cobas® 6000 Modular Analyzer, Roche Diagnostics, Basel, Switzerland, Europe). Internal control is ensured by daily checks provided by the manufacturer, while external control is ensured by the participation of interlaboratory circuits organized by the Sicilian Region.

A diet (free of cheese, yogurt, industrial sweets, dried fruit, fruit preparations, canned food, fizzy drinks) was prescribed to workers during the entire sampling period in order not to influence the study results. T,t-MA is also a metabolite of sorbic acid, which is a substance naturally contained in some foods or used as a food preservative.

### Statistical Analysis

A preliminary statistical analysis of the data was performed using STATA 12 software (41). Comparisons were performed with Student's *t*-test for independent samples (smokers vs. non-smokers) and for paired samples too (beginning of the shift work vs. end of the shift work). The criterion for significance was set at *p* < 0.05. For descriptive analysis (media, median, and range), results are presented as μg/g creatinine.

## Results

### Study Population

The adherence rate to the study was 100%. Table 1 shows the general characteristics of the study population. The overall sample consisted of 43 male port workers ranged in age from 21 to 64 years and with a mean working life of 19.63 years. Workers were divided into two groups: 15 smokers (average age 48 years old and mean working life of 21.4 years) and 28 non-smokers (average age of 47.07 years old and mean working life of 18.68 years). The comparison between the group of smokers and non-smokers did not show significant differences for age, BMI, and years of service. With regards to smokers, the average number of cigarettes smoked per day was 16.33, and the mean number of years as a smoker was 29.73.

**Table 1:** General characteristics of study population, n°43.

	**Overall (N^**°**^43)**	**Smokers (N^**°**^15)**	**Non-smokers (N^**°**^28)**
**Age (Years)**			
Mean ± SD	45.44 ± 9.93	48 ± 10.13	47.07 ± 9.73
Median	47	49	44
Range	21–64	21–64	24–62
**BMI (Kg/m**^**2**^**)**			
Mean ± SD	21.61 ± 1.87	21.69 ± 1.81	21.57 ± 1.93
Median	21.8	21.8	21.7
Range	17.6–25.1	18.6–25.1	17.6–25.1
**N°** **Cigarettes/day**	N/A		N/A
Mean ± SD		16.33 ± 6.4	
Median		15	
Range		10–30	
**N°** **of Years as Smoker**	N/A		N/A
Mean ± SD		29.73 ± 8.88	
Median		29	
Range		6–46	
**Length of service—years**			
Mean ± SD	19.63 ± 9.55	21.4 ± 10.45	18.68 ± 9.09
Median	20	22	19
Range	2–41	2–41	2–41

### Biological Monitoring

The urinary t,t-MA was detected as a marker of the internal dose of benzene exposure. Table 2 shows the results of the analysis of t,t-MA urinary excretion at the beginning and at the end of the work shift in the overall sample of workers, divided for smoking and non-smoking habit. The results were subdivided by smoking habit because reports in literature have demonstrated that cigarette smoke influences urinary excretion of this metabolite (42). In the overall sample, the median value of t,t-MA urinary excretion was 79.8 and 102.7 μg/g creatinine at the beginning and the end of the work shift, respectively. At the beginning of the work shift, the lowest concentration of t,t-MA was 17.5 μg/g creatinine in non-smokers, and the highest was 231 μg/g creatinine in smokers. At the end of the work shift, the lowest concentration of t,t-MA was 23.9 μg/g creatinine in non-smokers, and the highest was 325.4 μg/g creatinine in smokers.

**Table 2:** T,t-muconic acid urinary excretion.

	**Overall (N^**°**^43)**	**Smokers (N^**°**^15)**	**Non-smokers (N^**°**^28)**
**T,t-muconic acid urinary excretion**			
**Beginning of shift work**			
**Mean**			
μg/g creat	81.94 ± 46.28	116.59 ± 50.03	63.39 ± 31.78
**Median**			
μg/g creat	79.8	90.5	61.45
**Range**			
μg/g creat	17.5–231	52.3–231	17.5–124.9
**T,t-muconic acid urinary excretion**			
**End of shift work**			
**Mean**			
μg/g creat	117.52 ± 58.91^*^	153.19 ± 65.64	98.41 ± 45.59^**^
**Median**			
μg/g creat	102.7	128.2	89.5
**Range**			
μg/g creat	23.9–325.4	87.4–325.4	23.9–189.6

* *p = 0.002*;

** *p = 0.003: comparison between mean t,t-MA urinary excretion at the beginning of the shift work and at the end of the shift work*.

**Figure 1:**
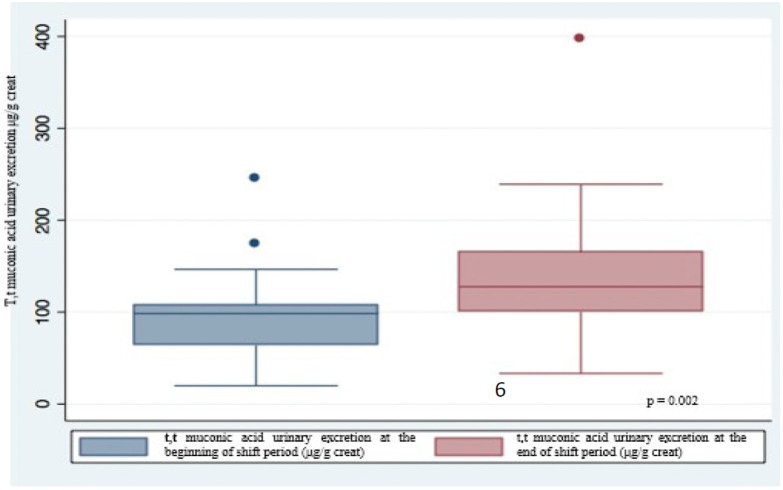
Urinary excretion of t,t-muconic acid at the beginning and at the end of the shift work.

**Figure 2:**
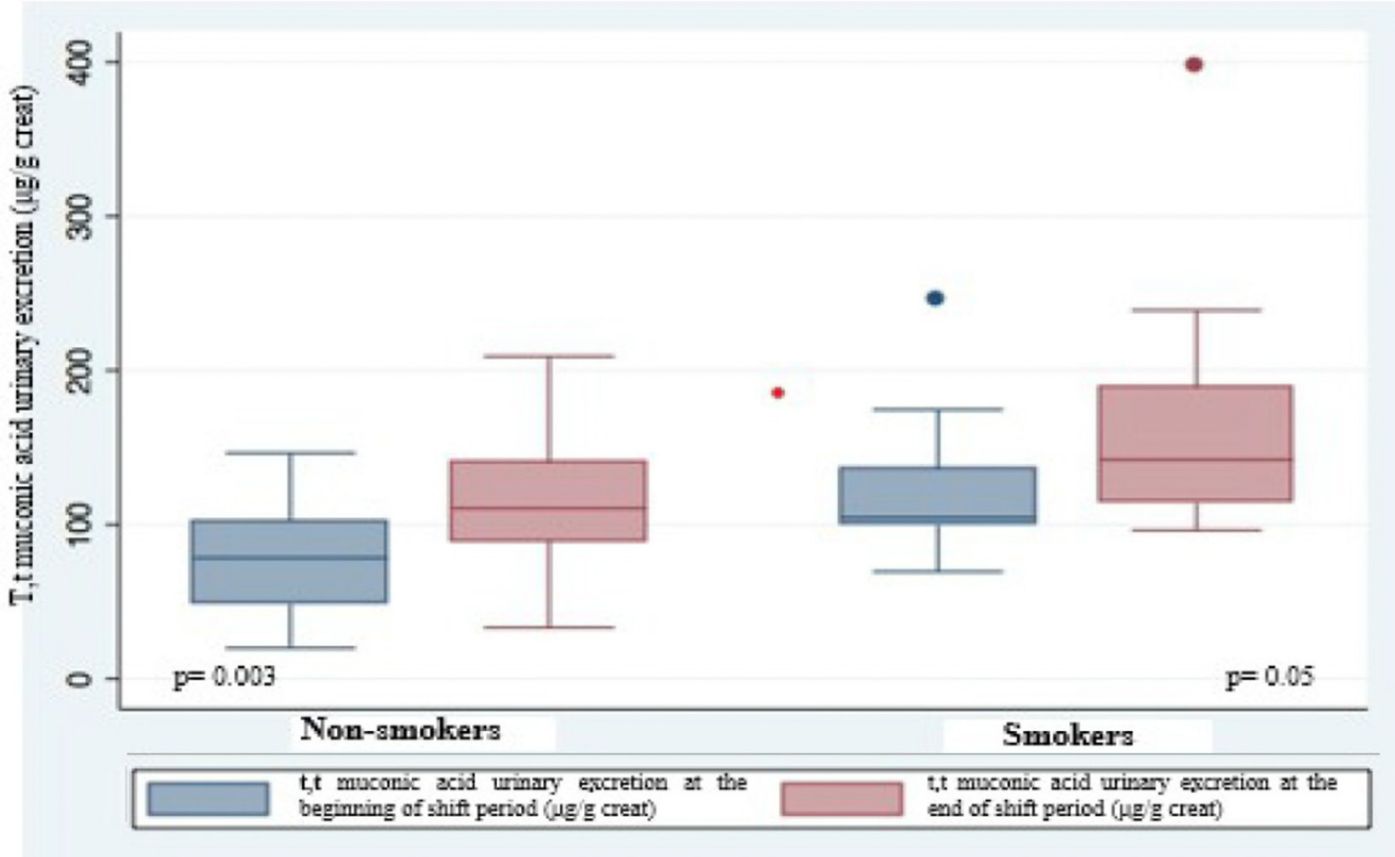
T,t-muconic acid urinary excretion in smokers and non-smokers.

Urinary excretion of t,t-MA was always higher at the end of the work shift than at the beginning with significant difference (*p* = 0.002) (Figure 1); this difference is stronger in non-smokers (*p* = 0.003) than in smokers (*p* = 0.05) (Figure 2). In smokers, median t,t-MA urinary excretion is higher than non-smokers both at the beginning (90.5 μg/g creatinine vs. 61.45 μg/g creatinine) and at the end of the work shift (128.2 μg/g creatinine vs. 89.5 μg/g creatinine).

## Discussion

According to the International Labour Organization (ILO), the main health hazards related to port operations are, among others, fumes, dust, and exposure to hazardous chemical substances (29). These substances can cause serious health effects in exposed individuals (43). Despite the multitude of harmful substances that may be present in port areas, there are limited pieces of information about biological monitoring and implications of chemical risk in port workers.

The results of our study showed that the levels of t,t-MA in urine samples taken from all workers (smokers/non-smokers) at the end of the shift had significantly higher values compared with those measured at pre-shift. The median value of t,t-MA urinary excretion in smoking workers is higher both at the beginning and at the end of the shift than in non-smoking workers. The urinary concentration of t,t-MA never exceeded the BEI of ACGIH 500 μg t,t-MA/g creatinine, in both smokers and non-smokers.

In agreement with our results, a recent study by Forsell et al. (44) explores benzene exposure in deck crews on tankers investigating the correlations between benzene exposure and benzene in alveolar air, benzene in urine, and t,t-MA in urine. They found that the average 4-h benzene exposure level for those exposed was 0.45 mg/m^3^ and for those non-exposed 0.02 mg/m^3^. All the biomarkers were significantly higher in post-shift samples among exposed and correlated with the exposure level.

Furthermore, Davenport et al. (45) conducted a study in an attempt to quantify short-term exposure levels to Coast Guard personnel performing routine inspection activities aboard commercial tank barges carrying gasoline. A total of 43 personal and 68 area samples were analyzed for benzene. Although none of the personal samples met or exceeded proposed or established short-term exposure standards, many of the area sampling results indicated that a significant risk of acute exposure exists in the vicinity of valves, pressure lines, and connections. Fakhrinnur et al. (38) measured t,t-MA levels in a group of 33 service station workers and found a significant correlation between the duration of filling the fuel and the level of t,t-MA in urine (*p* = 0.000). Also, unlike our results, seven workers had urine tt-MA levels that exceeded the value of BEI set by ACGIH (500 μg/g creatinine).

In our study, biomonitoring of benzene exposure was assessed by measuring one of its urinary metabolites, the t,t-MA, a reliable biomarker for low-level benzene exposure. T,t-MA is an indicator used for routine biological monitoring, mainly due to the analytical method for determining its concentration (HPLC-UV), practicable in all industrial and environmental toxicology laboratories (46, 47).

Among factors affecting urinary t,t-MA detection is the sorbic acid (additive in food, cosmetics, and pharmaceuticals), and therefore, workers were subjected to an elimination diet during the sampling period. In humans, after ingestion of a single dose of 447 mg sorbic acid and, during 2-day trials, ingestion of three doses of 1 mg sorbic acid/kg body weight, it was found that about 0.05–0.51 and 0.15–0.34%, respectively, of the dose was converted into t,t-MA. Weaver et al. found that in subjects who consumed two sorbic acid–preserved foods, a great increase in t,t-MA urinary concentrations was observed with individual peaks ranging as high as 705 μg/g creatinine (48, 49).

Our study has some limitations. We have no information about the use of personal protective equipment (PPE) and a not very large sample. In addition, we do not have data relating to the actual duration of the refueling activity during each single working shift and during the entire working week, because this activity is variable depending on the demand. On the other hand, our study also has strengths. The main one is the systematic biological monitoring, carried out through the determination of t,t-MA in morning (first urination of the day) and evening urine samples, which allowed us to obtain valuable information about the exposure to benzene of each port worker.

In conclusion, the increase in urinary excretion of t,t-MA from the beginning to the end of work shift in exposed non-smoking workers showed that the role of inhaled benzene at fuelling stations in a tourist port can be relevant. These findings are important considering the forthcoming OEL reduction. If, in fact, in the past, port workers were considered exposed to low doses of benzene, almost always below the OELs, in the near future, this will no longer be true. As a consequence, it will become increasingly important to monitor benzene exposure in order to keep it below new OELs and avoid adverse effects on workers' health.

On the basis of these results, we encourage the use of PPE in the fuelling area of ports in order to minimize exposure to benzene. In addition, port workers should undergo pre-placement and periodic medical examinations in order to identify health problems caused by exposure to benzene.

## Data Availability Statement

The datasets generated for this study are available on request to the corresponding author.

## Author Contributions

CL, EC, VR, and LV: conceptualization. CL, EC, and VR: investigation and data curation. GF and DC: formal analysis and methodology. LV: supervision. LD, AC, FM, EC, SS, and FB: writing—original draft. LV, LD, AC, FM, EC, SS, and FB: writing—review and editing. All authors contributed to the article and approved the submitted version.

## Conflict of Interest

The authors declare that the research was conducted in the absence of any commercial or financial relationships that could be construed as a potential conflict of interest.
